# Auditory Cortex Distinguishes between Spontaneous and Sound-Evoked Movements

**DOI:** 10.1523/JNEUROSCI.1874-25.2026

**Published:** 2026-05-29

**Authors:** Paul Zimmer-Harwood, Samuel Picard, Andrew J. King, Johannes C. Dahmen

**Affiliations:** Department of Physiology, Anatomy and Genetics, University of Oxford, Oxford OX1 3PT, United Kingdom

**Keywords:** auditory, brain state, imaging, mouse, movement, somatosensory

## Abstract

Activity in sensory cortex is influenced by multiple factors beyond sensory input, including body movements, neuromodulatory signals, and internal states such as arousal. Focusing on brief whisking bouts that occur independently of locomotion and which are a reliable indicator of cholinergic and noradrenergic input to the cortex, we investigated how these factors shape activity in the auditory cortex. By tracking the movements of individual whiskers in male and female mice, we observed that whisking events co-occur with subtle whole-body “twitches” and are followed by a dilation of the pupil that scales in size with the whisker movement. Although this behavior occurred spontaneously, near-identical whisker movements could also be elicited by pure tones. Whisking was reliably triggered by moderately loud, 80 dB SPL, tones at frequencies within the most sensitive region of the mouse’s hearing range, with measurable whisker movements following tone presentation at levels as low as 50 dB SPL. Tone-triggered whisking was sensitive to the recent stimulus history but did not habituate over longer time periods. The activity of a subset of neurons in the auditory cortex was significantly modulated in relation to spontaneous whisking events. Surprisingly, many of those neurons did not respond or responded differently when whisking was sound triggered, suggesting that the context and the underlying driver of a body movement determine whether and how it modulates auditory cortical activity.

## Significance Statement

Neural activity in sensory cortices is strongly shaped by movements and internal states, but the drivers of these influences remain unclear. We show that brief whisker twitches in mice, which are linked to arousal-related neuromodulatory input, are represented in the auditory cortex. Surprisingly, nearly identical whisker movements can also be triggered by sounds, yet many neurons respond differently depending on whether whisking is spontaneous or sound evoked, revealing that the same outward behavior can engage distinct neural mechanisms depending on its origin. Our findings highlight the importance of considering the context and cause of movements when interpreting brain activity, with broad implications for studies of sensory processing and behavior.

## Introduction

Auditory cortical activity reflects not only the encoding of acoustic information but also the complex interplay of various internal and behavioral states, neuromodulation, and motor signals. Ongoing variations in neural activity can reflect different brain states encompassing arousal, motivation, emotions, and homeostatic needs (McCormick et al., 2020; Flavell et al., 2022). In mice, arousal fluctuations manifest as cortex-wide changes in excitation, and externally observable indicators—such as pupil diameter and body movements—have been identified as useful markers of these fluctuations (McGinley et al., 2015a,b; Musall et al., 2019; Stringer et al., 2019; Collins et al., 2023).

The neuromodulators acetylcholine (ACh) and noradrenaline (NA), in particular, have been implicated in controlling arousal-related changes in cortical activity via broadly projecting axonal pathways originating in the basal forebrain and locus ceruleus, respectively. Cortical ACh and NA activity tracks changes in pupil diameter (Nelson and Mooney, 2016; Reimer et al., 2016; Larsen et al., 2018; Collins et al., 2023) and behavioral engagement, indexed as locomotion, licking, or whisking (Eggermann et al., 2014; Harrison et al., 2016; Collins et al., 2023). Furthermore, activating the locus ceruleus can promote locomotion (Carter et al., 2010), while activating the mesencephalic locomotor region increases both locomotor activity and, via its projection to the basal forebrain, cortical gain (Lee et al., 2014).

Body movements can therefore reflect the effects of arousal and arousal-related neuromodulation on the cortex (Reimer et al., 2016; Collins et al., 2023). However, motor signals also have a more direct and specific function, particularly within the auditory cortex, where access to an efference copy (Von Holst and Mittelstaedt, 1950) of relevant motor commands is thought to be crucial for distinguishing self-generated from external sounds, as well as for detecting errors in self-generated sounds. Integrating motor and auditory signals is therefore important for vocal learning (Eliades and Wang, 2008; Keller and Hahnloser, 2009) but also other sound-guided behaviors (Schneider et al., 2018). Projections from secondary motor cortex (Nelson et al., 2013) have been implicated in the suppression of auditory cortical activity during movement (Schneider et al., 2014). Furthermore, movement-related inputs can modulate activity as early as the cochlear nucleus in the brainstem (Kanold and Young, 2001; Singla et al., 2017), as well as later subcortical processing stages (Suga and Schlegel, 1972; Gruters and Groh, 2012; Zhou et al., 2014; McGinley et al., 2015a; Williamson et al., 2015; Bigelow et al., 2019; Chen and Song, 2019; Yang et al., 2020; Efron et al., 2025), indicating that motor-auditory integration can take place throughout the auditory pathway.

An additional complication is provided by the often bidirectional relationship between motor output and sound. While body movements can produce sounds that potentially need to be registered as self-generated, sounds can also trigger body movements. This is most obvious when loud sounds elicit a startle (Koch, 1999) or flight (Xiong et al., 2015) response but also evident in subtler movements that can be triggered by weaker, especially broad spectrum, sounds (Meyer et al., 2018; Bimbard et al., 2023; Clayton et al., 2024; Olsen and Hasenstaub, 2025; Bigelow et al., 2026).

Here, we investigate the interplay between body movement, brain state, and auditory cortical activity by focusing on brief whisking bouts, or twitches, occurring outside periods of locomotion and which are thought to indicate a spike in cortical neuromodulatory activity (Collins et al., 2023). Using high-speed videography, we found that these whisking events are very tightly coupled to changes in pupil diameter and that their occurrence is represented by the activity of a subset of neurons in the auditory cortex. While these events occur spontaneously, we also found that near-identical whisker movements can be triggered by pure tones spanning certain frequency-level combinations. Surprisingly, many neurons that were modulated by spontaneous whisking events responded differently to sound-triggered whisking, suggesting that the capacity of body movements to modulate auditory cortical activity depends on the context in which they occur and the underlying drivers of those movements.

## Materials and Methods

### Animals

**Figure 1.**
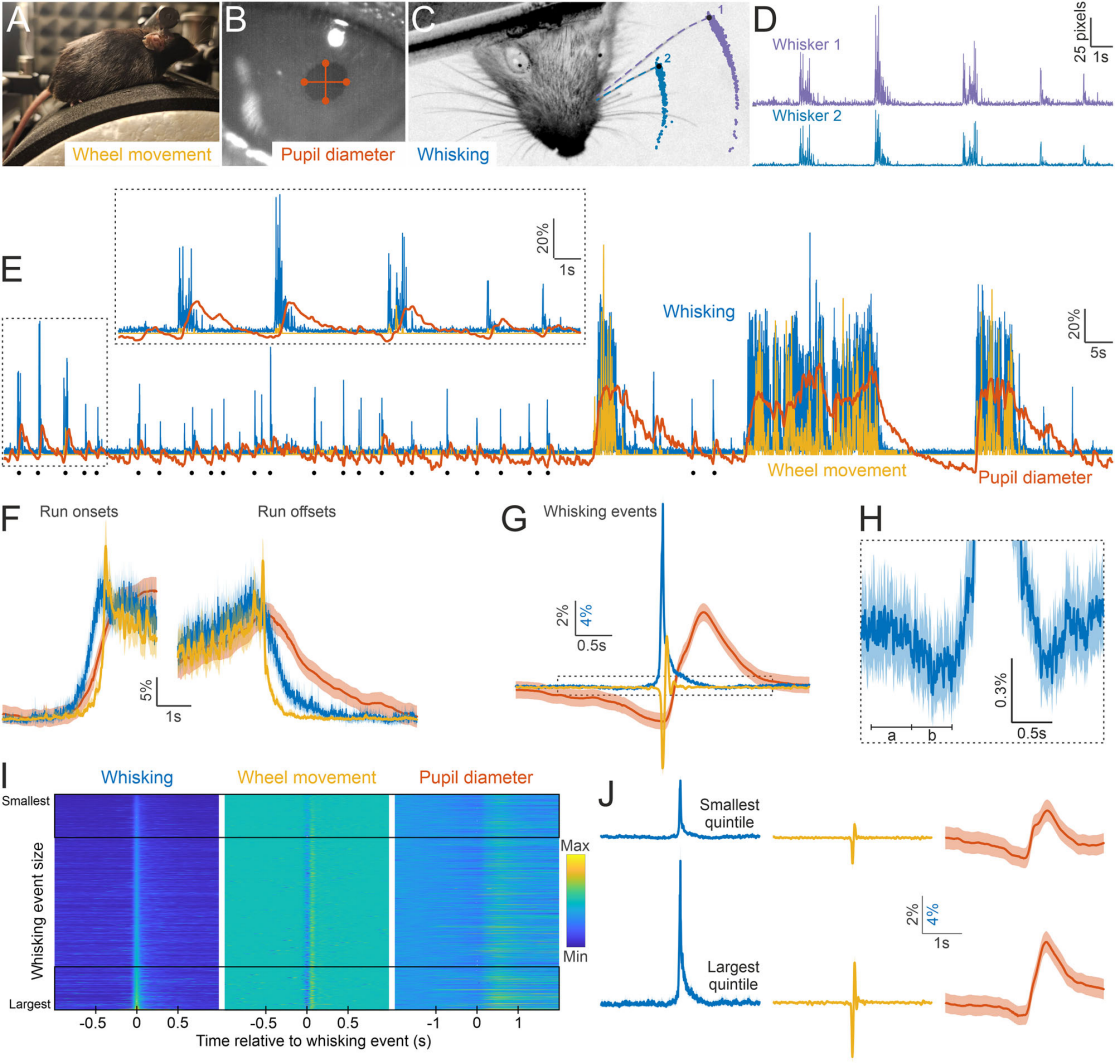
Relationship between whisking, other behaviors and measures of behavioral state. ***A***, Head-fixed mice were placed on a polystyrene wheel to measure locomotion speed as well as more subtle body movements not associated with running or walking. ***B***, ***C***, Video recordings were made to measure the pupil diameter and the movements of individual whiskers. Dots in ***C*** indicate tip positions of two whiskers measured in a 17-s-long example video segment (Movie 1). ***D***, Frame-by-frame change in tip position of the two whiskers from ***C*** measured in the same example video segment. ***E***, Whisking, wheel movement (i.e., locomotion), and pupil diameter traces measured over a 200-s-long set of example recordings. The inset corresponds to the segment shown in ***D***. The whisker movement trace represents the average movement of the two whiskers tracked during this session. All variables are normalized by and expressed as a percentage of the session maxima. Dots underneath the traces indicate the timing of whisking events, i.e., whisking that takes place outside of periods of locomotion (and not within 10 s after a period of locomotion). ***F***, Mean whisking, pupil diameter, and wheel movement traces aligned to the onset (left, *n* = 111) or offset (right, *n* = 110) of running. For simplicity, ***E*** and ***F*** show absolute wheel movement, i.e., irrespective of the wheel's direction of rotation. ***G***, Mean whisking, pupil diameter, and wheel movement traces aligned to the whisking event (*n* = 743 events). Here, a downward deflection in the wheel trace indicates a backward movement of the wheel. The blue percentage value indicates the scale of the whisking trace, and the black one applies to the other parameters. Note the difference in scale between ***G*** and ***F***. ***H***, Expanded segment of the whisking trace replotted from ***G***. A significant drop in median whisking activity was observed between consecutive 500 ms time segments “a” and “b” preceding the whisking event. ***I***, Heatmaps of all whisking, pupil, and wheel movement traces aligned to and ordered according to the size of the corresponding whisking event. Note the difference in *x*-axis scale. ***J***, Mean whisking, pupil, and wheel traces for the smallest (bottom quintile, *n* = 148, top rectangle in ***I***) and largest (top quintile, *n* = 148, bottom rectangle in ***I***) whisking events. Shading in ***F***–***H*** and ***J*** indicates 95% confidence intervals.

**Figure 2.**
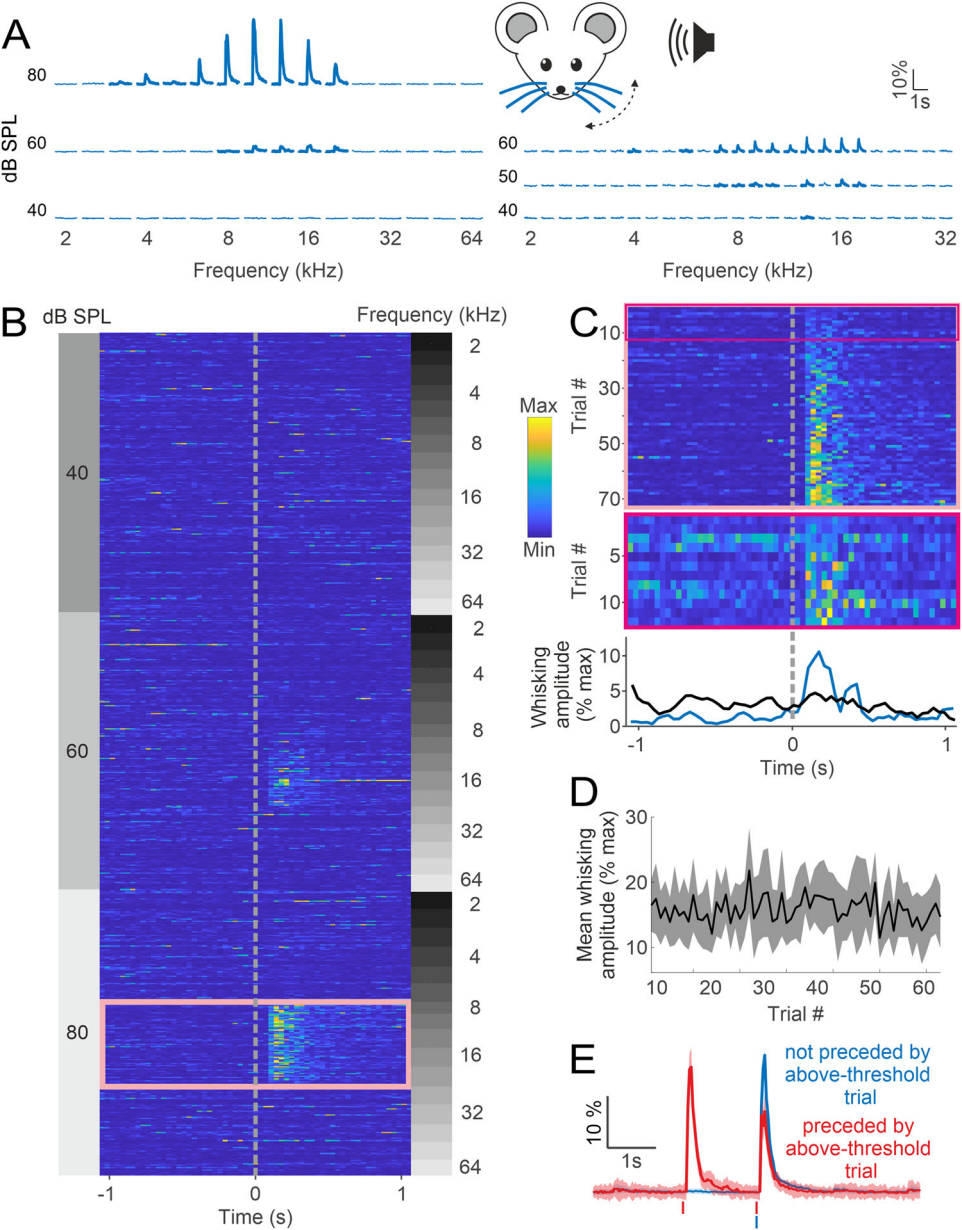
Pure tones trigger whisking. ***A***, Left, Mean sound-triggered whisking as a function of pure tone frequency and level for three mice. Thick blue traces indicate frequency-level combinations that triggered a statistically significant increase in whisking (*p* < 0.05, Wilcoxon signed rank test, *n* = 234–254 sound presentations per frequency-level combination across mice and sessions; some sessions were conducted with mice on the polystyrene wheel so sound presentations occurring during locomotion periods were excluded). ***A***, Right, Same as ***A***, left, for two different mice presented with lower level tones and with the mice positioned in an acrylic body tube, *n* = 120 sound presentations per frequency-level combination across mice and sessions. ***B***, Whisking aligned to sound onset (dashed gray line) for all trials from one recording session, with trials ordered according to sound level and frequency. ***C***, Top, Whisking aligned to sound onset (dashed gray line) showing an expanded view of all trials from one recording session in which 80 dB SPL tones of frequencies that were particularly effective in triggering whisking were presented (pink rectangle in ***B***, *p* < 0.001, referred to as “above-threshold trials”). For this recording session, the effective tones spanned frequencies from 8 to 20.15 kHz. Trials are ordered according to the amplitude of the whisker movement. ***C***, Middle, The 12 trials with the smallest whisker movement from ***C***, top, replotted with a different color scale; all but four trials appeared to include sound-locked whisker movements. ***C***, Bottom, The average whisking trace from trials 1–4 (black) and the trace from trial 5 (blue). ***D***, Mean sound-triggered whisking amplitude (as a percentage of the maximum value in each session) across 14 recording sessions (3 mice) as a function of trial number. Only above-threshold trials were selected. ***E***, Mean whisking amplitude in above-threshold trials that either were (red, *n* = 191) or were not (blue, *n* = 1,288) themselves preceded by an above-threshold trial. Vertical lines indicate onsets of above-threshold sounds. Shaded areas in ***D*** and ***E*** indicate 95% confidence intervals.

All experiments were approved by the Committee on Animal Care and Ethical Review at the University of Oxford and were licensed by the UK Home Office (Animal Scientific Procedures Act, 1986, amended in 2012). The data shown in Figure 1 were obtained from three male B6;129S-*Gt(ROSA)26Sor^tm95.1(CAG-GCaMP6f)Hze^*/J (Ai95D, JAX 024105, Jackson Laboratories) × *Slc32a1^tm2(cre)Lowl^*/J (JAX 016962); the data shown in Figure 2*A*, right, were obtained from two female Ai95D × B6.Cg-Tg(*Camk2a*-cre)T29-1Stl/J (JAX 005359); and the remaining data from three male B6;DBA-Tg(tetO-GCaMP6s)2Niell/J (JAX 024742) × B6.Cg-*Igs7^tm94.1(tetO-GCaMP6s)Hze^*Tg(Camk2a-tTA)1Mmay/J (JAX 024115) mice. All mice were ∼3 months old during data collection. They were maintained on a 12 h light/dark cycle and were housed at 20–24°C with a relative humidity of 45–65%.

### Surgeries

For all surgical procedures, mice were premedicated with intraperitoneal injections of dexamethasone (Dexadreson, 4 mg), atropine (Atrocare, 1 mg), and carprofen (Rimadyl, 0.15 mg) before being anesthetized with isoflurane (1.5–2%) and administered with buprenorphine (Vetergesic, 1 ml/kg) postoperatively. Mice were then placed in a stereotaxic frame (Model 900LS, David Kopf Instruments), and their body temperature was kept constant at 37°C by the use of a heating mat and a DC temperature controller in conjunction with a temperature probe (FHC). Head bar implantation for mice that only underwent behavioral assessment without imaging involved removing a flap of skin of ∼1 cm diameter over the center of the skull and using dental acrylic (Super-Bond C&B, Sun Medical) to embed a custom steel post and to seal the exposed skull. Imaging window implantation involved the removal of slightly more skin on the right side of the skull, as well as partial removal of the right temporal muscle to make room for a circular 4 mm craniotomy covering the auditory cortex. A 4-mm-diameter glass coverslip that had been glued to a ∼1-mm-tall steel cylinder with 0.5 mm wall thickness was inserted into this craniotomy. The cylinder allowed us to press the glass window gently onto the brain (in order to minimize brain movement during experiments) and was then glued (Pattex Ultra Gel, Henkel) to the edges of the skull. Mice were allowed to recover for at least 1 week before the first recording session.

### Whisker, pupil, and locomotion recording

A high-speed camera (DMK 37BUX287, The Imaging Source) recording at 200 (Fig. 1) or 30 (all other data) frames per second was used to capture the movement of individual whisker tips. Two infrared illuminators (LIU780A, Thorlabs) were placed right and left in front of the mouse and angled inwards, such that light could be reflected upward by the whiskers toward the down-facing camera to achieve short camera shutter speeds. A second camera (DMK 23UV024, The Imaging Source) was used to capture the pupil. Mice were either placed in a transparent acrylic body tube or on a freely rotating polystyrene wheel, coated with a layer of isolation foam to attenuate the noise caused by the steps of the mouse. When placed on the wheel, the angular motion of the running wheel was recorded using an optical mouse (Logitech G500) and captured using LabVIEW (LabVIEW 2017, National Instruments) in order to measure the animals' locomotion speed. All experiments were performed in a sound-attenuated chamber. Recording sessions not involving calcium imaging were done in moderately lit conditions rather than complete darkness to avoid permanent dilation of the pupil.

### Sound presentation

Pure tones of 200 ms length with 10 ms raised cosine onset and offset ramps were presented randomly interleaved from 2 to 64 kHz in 1/3 octave steps, at levels of 40, 60, and 80 dB SPL and with an interstimulus onset interval of 1.5 s. The data shown in Figure 2*A*, right, were collected with pure tones presented randomly interleaved from 2 to 32 kHz in 1/6 octave steps, at levels of 40, 50, and 60 dB SPL and with an interstimulus onset interval of 1 s. The stimuli used for widefield imaging were sinusoidally amplitude-modulated (SAM) tones with a duration of 500 ms and a modulation frequency of 10 Hz (100% modulation depth) presented either at 55 or 65 dB SPL and four frequencies: two low frequencies (4.00 and 5.04 kHz) and two high frequencies (25.4 and 32.0 kHz). SAM tones were presented sequentially with a 3 s interstimulus onset interval and repeated 10–15 times. All stimuli were generated at a 192 kHz sampling rate with custom-written LabView programs (National Instruments) and a 16 bit sound card (Xonar X7, Asus), amplified (Portable Ultrasonic Power Amplifier; Avisoft Bioacoustics) and presented through an ultrasonic free-field electrostatic speaker (Avisoft Vifa), placed centrally ∼10 cm from the animal's head. Sound presentation equipment was calibrated to ensure a flat (±2 dB) response across all frequencies. Image acquisition and stimulus presentation were synchronized via a NI-DAQ I/O device (USB-6501, National Instruments).

### Calcium imaging

We performed widefield calcium imaging as described previously (Weissenberger et al., 2019) to identify auditory cortical subfields using a 340M-GE CCD camera (Thorlabs), TL2x-SAP objective (Thorlabs), and 470 nm LED (M470L4, Thorlabs), all of which were fitted to the body of a rotating Bergamo II microscope (Thorlabs). Two-photon calcium imaging was performed in the primary auditory cortex (A1) and the anterior auditory field (AAF), 150–250 µm from the brain surface (cortical layers 2/3) using a rotating multiphoton laser-scanning microscope (Bergamo II, Thorlabs) fitted with a 20×/1.00 immersion objective (Olympus). Excitation light (940 nm) was emitted from a femtosecond laser (Chameleon Discovery, Coherent) at ∼1,300 mW and scanned onto the brain with an 8 kHz resonant scanner and a galvanometric scan mirror. Laser power as measured under the objective was typically 45–70 mW. ScanImage 2018 (Vidrio Technologies) was used to control the microscope. The resonant scanner was used in bidirectional mode, enabling the acquisition of 512 × 512 pixel frames in a 600 × 600 µm field-of-view at a rate of ∼30 Hz. The sound level generated by the resonant scanner was <40 dB SPL near the mouse's head. Rigid and nonrigid image registration, segmentation, neuropil, and signal extraction were performed using the Python version of suite2p (Pachitariu et al., 2016). Neuropil correction was carried out using a coefficient of 0.7 (Kerlin et al., 2010) and calcium Δ*F*/*F* traces were obtained by using the median over the entire fluorescence trace (excluding fluorescence values below the 10th and above the 70th percentile) as the *F*_0_.

### Data analysis

We measured changes in pupil diameter and identified whisker movements by using a Python implementation of DeepLabCut (Mathis et al., 2018) to track four points on the pupil's rim (Fig. 1*B*) and the positions of the tips (Fig. 1*C*) of 1–3 whiskers in each recording. Neural network training (ResNet50) was done over 1 million epochs based on ∼120 hand-labeled video frames per animal, and final performance was evaluated to have an average Euclidean distance error of <7 pixels for all recordings. If necessary, additional frames were hand-labeled and training commenced for a second iteration. A unique network was trained for every mouse to identify individual whiskers. Further data analysis was carried out in Matlab (MathWorks). The frame-by-frame change in whisker tip position was averaged across all whiskers tracked for a given session to obtain a single whisker movement trace for each recording. Pupil diameter was defined as the average distance between horizontally and vertically aligned pairs of points. Except for the analysis illustrated in Figure 1*F*, data recorded within periods of locomotion or up to 10 s after the end of a locomotion period were excluded from analysis. In order to identify whisking events, we applied the Matlab “findpeaks” function to a smoothed (Gaussian of 100 ms width) version of the whisker movement trace. Whisking events occurring within 2 s of another whisking event were excluded from analysis.

Locomotion was defined as a wheel speed exceeding 3 cm/s. Facial motion energy was defined as the summed, pixelwise, absolute frame-by-frame difference in a region of interest covering the whiskerpad. Unless stated otherwise, pupil diameter as well as whisker and wheel movements are plotted as a percentage of the maximum value recorded for each variable in the same session. During sessions involving two-photon calcium imaging, pure tones were presented at levels of 40, 60, and 80 dB SPL. A whisking event was categorized as spontaneous if it did not occur within 1 s after the onset of a tone with a frequency-level combination that causes a significant (*p* < 0.05, Wilcoxon signed rank test) increase in mean whisker movement from the 500 ms before sound onset to the 300 ms after sound onset. This approach excluded, as desired, even frequency-level combinations that cause very small increases in whisking. To find whisking events that were sound triggered, we applied very stringent criteria (*p* < 0.001, only 80 dB SPL sounds) to identify frequency-level combinations that reliably trigger whisking. Whisking events that occurred within 1 s of the onset of a tone with such a frequency-level combination were considered sound triggered. Neurons were considered to be modulated by spontaneous (sound-triggered) whisking if they exhibited a statistically significant difference (*p* < 0.001, Kruskal–Wallis test) in fluorescence among the 70 frames (from 20 frames before the whisking event peak to 50 frames after) surrounding spontaneous (sound-triggered) whisking events.

To assess whether individual neurons distinguish between spontaneous and sound-triggered whisking, we calculated receiver operating characteristic (ROC) curves for the distributions of neural activity measured around spontaneous and around sound-triggered whisking events. An ROC curve was calculated separately for each time point (imaging frame) from 100 frames before the whisking event to 100 frames after the whisking event, and the corresponding area under the curve (AUC) was considered significant if it exceeded the AUC of a control ROC curve obtained from shuffled (100 iterations) distributions by ±3 standard deviations (Gilad et al., 2020).

To compare neural responses to sounds that occurred when the mouse was locomoting with those that occurred when the mouse was stationary, and to compare responses that occurred when the mouse was whisking with ones that occurred when it was not whisking, we considered only responses to tones of frequency-level combinations that did not trigger significant whisking. We then selected neurons that exhibited a significant difference in response (one-way ANOVA, *p* < 0.001) among the remaining frequency-level combinations. From each of those neurons, we selected the frequency-level combinations with a mean response of ≥1.5 standard deviations above the overall mean response for that neuron. We then identified stimulus presentations that occurred when the mouse was locomoting or stationary and when the mouse was whisking or not whisking (periods of locomotion were excluded from the comparison of whisking vs not whisking). Whisking here was defined as periods in which a smoothed (Gaussian of 1 s width) whisker movement trace exceeded 5% of the maximum whisker movement recorded in that session. Periods below that threshold were defined as “not whisking.” If we could identify at least five stimulus presentations during locomotion and at least 5 matching (i.e., of the identical frequency-level combination) stimulus presentations that occurred during stationary periods, the neuron was included in the dataset to evaluate the effect of locomotion on sound-evoked activity. Analogously, if we could identify at least five stimulus presentations that occurred when the mouse was whisking and at least five matching stimulus presentations that occurred when the mouse was not whisking, the neuron was included in the dataset to evaluate the effect of whisking on sound-evoked activity. Note that the combination of these selection criteria substantially reduced the number of neurons available for this analysis, especially with respect to locomotion.

## Results

Our aim was to investigate the influence of whisking on the activity of neurons in the mouse auditory cortex. To this end, we first sought to establish how whisking relates to other behaviors and measures of brain state. We placed head-fixed mice on a freely rotating polystyrene wheel inside a moderately lit, sound-attenuated chamber and recorded their locomotion speed as well as more subtle body movement not associated with locomotion. Simultaneously, we recorded (high-speed) videos of their whiskers as well as of one of their eyes (Fig. 1*A–C*). No part of the setup was within reach of the whiskers so the mice could whisk freely without the whiskers touching any objects; this is not thought to generate sounds in the absence of other body movements (Efron et al., 2025). Pupil diameter and whisking were measured by tracking 4 points on the pupil's rim (Fig. 1*B*) and the tips of up to 3 whiskers (Fig. 1*C*) using DeepLabCut (Mathis et al., 2018). High correlations between the movements of simultaneously tracked whiskers [median Pearson’s correlation coefficient = 0.96 ± 0.05 (interquartile range)] showed that the whiskers tend to move together (Fig. 1*D*), indicating that tracking a single whisker is sufficient to measure whisking. Furthermore, whisking was closely correlated with facial movements (Fig. S1*A*).

Consistent with previous work (Reimer et al., 2014), locomotion occurred together with vigorous whisking and dilation of the pupil (Fig. 1*E*). However, whisking [470 ± 619 ms (median ± interquartile range), *p* < 10^−13^, Wilcoxon signed rank test] and pupil dilation (265 ± 700 ms, *p* < 10^−12^, Wilcoxon signed rank test) tended to commence hundreds of milliseconds before locomotion (Fig. 1*F*). Whisking persisted for a similar duration after locomotion (625 ± 1,340 ms, *p* < 10^−14^, Wilcoxon signed rank test), while the pupil continued contracting for seconds afterwards (1,950 ± 1,665 ms, *p* < 10^−19^, Wilcoxon signed rank test; Fig. 1*F*). Whisking, however, also took place without locomotion and then typically occurred in brief bouts lasting a few hundred milliseconds (Fig. 1*E*, Movie 1). These brief whisking events were associated with a smaller dilation of the pupil than that occurring with locomotion (5.3 ± 5.8% vs 26.8 ± 16.2% increase in pupil diameter, *p* < 10^−50^, Wilcoxon rank sum test). While the mice did not run or walk, the polystyrene wheel's motion sensor typically picked up a brief back-and-forth movement, suggesting that these whisker movements occur as part of a whole-body “twitch.” Aligning all signals to the whisking event (Fig. 1*G*) illustrated that the whisking and body movement occur simultaneously and that the pupil dilation reaches its maximum approximately half a second later (540 ± 225 ms, *p* < 10^−40^, Wilcoxon signed rank test). A slight pupil contraction preceded the dilation, which was mirrored by a very small but statistically significant pre-twitch decrease in whisking (*p* < 10^−12^, Wilcoxon signed rank test; Fig. 1*H*). Apart from being closely aligned in time, these variables also scale in size (Fig. 1*I*,*J*). Larger whisking events were associated with larger body movements (*R* = 0.39, *p* < 10^−27^, Pearson’s correlation coefficient; Fig. S2*A*) and greater pupil dilation (*R* = 0.25, *p* < 10^−11^, Pearson’s correlation coefficient; Fig. S2*B*). Together, this suggests that all three signals—body and whisker twitching as well as pupil dilation—are externally observable signatures of a common change in brain state.

**Movie 1.** Video recording of mouse face and whiskers as well as one eye and the corresponding whisker movement, wheel movement, and pupil diameter traces. Video is played at half the speed of acquisition. Corresponds to data segment shown in Figure 1*D* and the inset of Figure 1*E*. [View online]

Next, we recorded whisker movements in mice in which we simultaneously monitored the activity of CaMKII alpha expressing excitatory neurons in layers 2/3 of the auditory cortex using two-photon calcium imaging. Recordings were made in the primary auditory cortex (A1, 10 sessions) and in the anterior auditory field (AAF, four sessions) after subfields had been identified using widefield calcium imaging, as described previously (Weissenberger et al., 2019). These recordings were carried out in the dark and with the mice placed inside a clear acrylic body tube for some sessions. Furthermore, we concurrently presented pure tones of varying frequencies and levels to investigate how whisking might interact with sound processing. Consistent with other recent reports we found that sounds can drive whisking. While other reports emphasized, in particular, the capacity of broadband sounds to elicit facial and body movements (Bimbard et al., 2023; Clayton et al., 2024; Olsen and Hasenstaub, 2025), we found that pure tones can also drive whisking provided their frequency lay within the most sensitive area of the mouse's hearing range between ∼3 and 20 kHz (Fig. 2*A*; Fig. S1*B,C*).

Pure tones caused significant increases in whisking when presented at a level of 50 dB SPL and potentially below (Fig. 2*A*). At 80 dB SPL, a greater range of tone frequencies triggered whisking on almost every trial (Fig. 2*B*,*C*). Furthermore, sound-triggered whisking events did not change in amplitude over the course of a session (*p* = 0.79, Mann–Kendall test; Fig. 2*D*), suggesting that mice do not habituate to the tones and that they remain effective at eliciting whisking even after dozens of presentations. However, the mean whisking amplitude was significantly smaller (11.9 ± 10.3% vs 15.5 ± 11.3%, *p* < 10^−6^, Wilcoxon rank sum test) in trials that were immediately preceded by another trial with a loud tone (Fig. 2*E*), suggesting that, at short time scales, sound-triggered whisking is modulated by stimulus history. Furthermore, there was a significant correlation between the mean whisking amplitude during the 2 s preceding a trial (only considering trials that were not themselves preceded by a loud tone in order to avoid the aforementioned effect of stimulus history) and the amplitude of the subsequent sound-triggered whisker movement, suggesting that higher baseline arousal favors stronger sound-triggered whisking (*R* = 0.25, *p* < 10^−19^). Whisking onset latency was estimated to be 67–100 ms, i.e., all mice started to whisk, on average, during the third video frame following sound onset.

**Figure 3.**
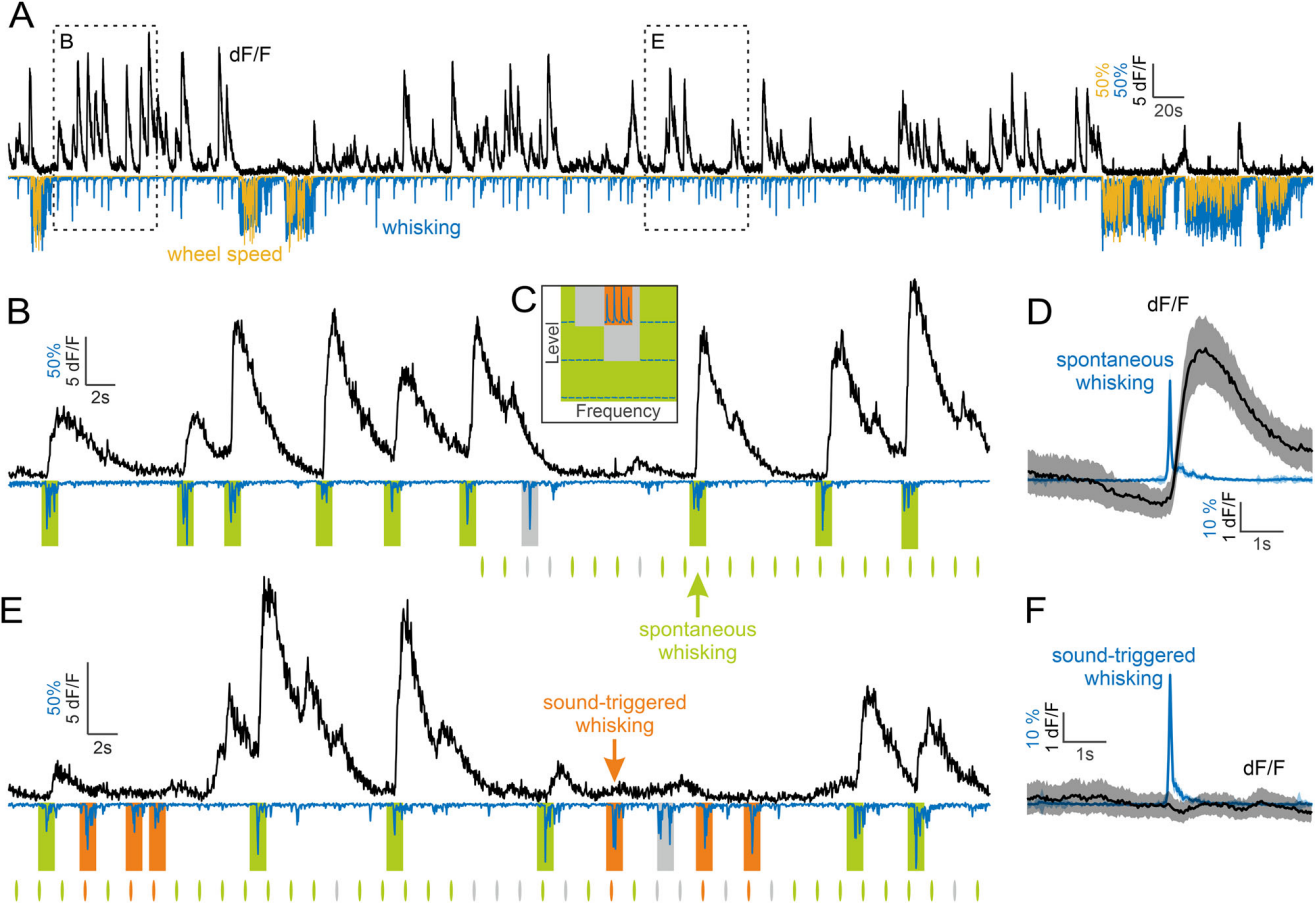
Relationship between whisking and neural activity for one example neuron in the auditory cortex. ***A***, Whisking (blue), wheel movement (yellow), and fluorescence responses (black) for one example neuron recorded in the auditory cortex. ***B***, Expanded illustration of the segment denoted with a small “B” in ***A***. Rectangles in ***B*** (and ***E***) indicate whisking events color coded according to how they were categorized. Vertical lines underneath the whisking trace indicate sound timings color coded according to whether a whisking event occurring immediately afterward would be considered spontaneous (green) or sound triggered (orange) or would be disregarded (gray). Each session started with a period of silence. Hence, the first sound was presented ∼20 s into the segment shown here. ***C***, Schematic illustration of how whisking events were categorized. A whisking event was categorized as spontaneous if it did not occur within 1 s of a tone of a frequency-level combination that for a given mouse causes a significant increase in whisking (green area, *p* < 0.05, Wilcoxon signed rank test). An event was considered sound triggered if it occurred within 1 s of an 80 dB SPL tone of a frequency that reliably triggered whisking (orange area, *p* < 0.001, “above-threshold trials”). Whisking events not falling into either of those categories were disregarded for further analysis (gray area). ***D***, Mean fluorescence response (black) for the same example neuron averaged across all spontaneous whisking events (blue) identified in this recording session (*n* = 78 events) and aligned to the whisking events. ***E***, Expanded illustration of the segment denoted with a small “E” in ***A***. ***F***, Mean fluorescence response (black) for the same example neuron averaged across all sound-triggered whisking events (blue) identified in this recording session (*n* = 53 events) and aligned to the whisking events. Shaded areas in ***D*** and ***F*** indicate 95% confidence intervals.

Given that only certain frequency-level combinations were associated with whisking (Fig. 2*A*), we were able to classify each whisking event as either sound triggered or spontaneous. A whisking event was considered spontaneous if it did not occur within 1 s of a tone of a frequency-level combination that causes a significant (*p* < 0.05, Wilcoxon signed rank test) increase in whisking. A whisking event was considered sound triggered if it occurred within 1 s of an 80 dB SPL tone of a frequency that reliably triggered whisking (*p* < 0.001, “above-threshold trials”). Whisking events not falling into either of those categories were disregarded for further analysis. We then identified neurons whose activity was truly coupled to whisking (rather than potentially sound driven by a tone/level combination that is also effective at triggering whisking) by considering exclusively the neural activity occurring around spontaneous whisking events (Fig. 3*A*–*D*).

**Figure 4.**
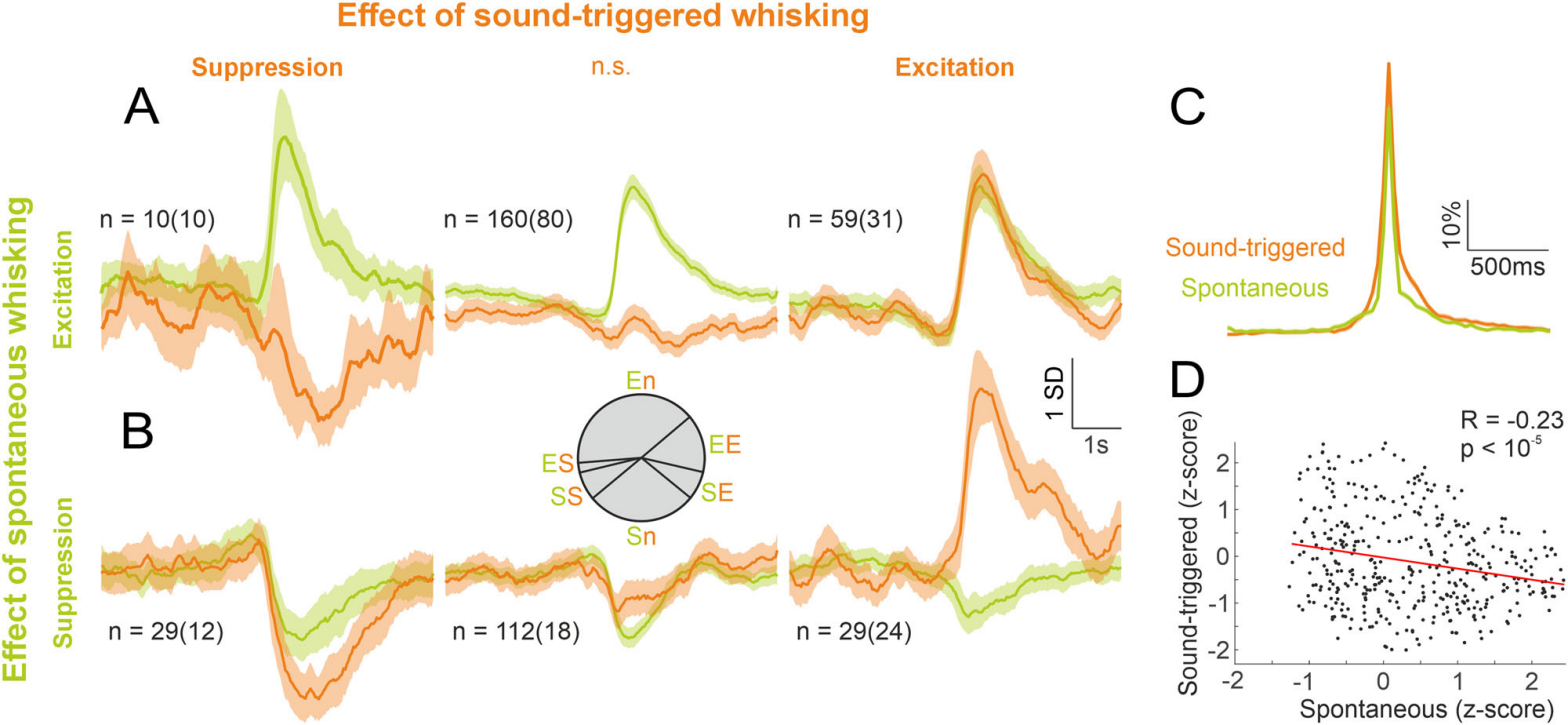
Categorizing whisking-sensitive neurons in the auditory cortex. ***A***, Mean fluorescence responses for neurons that were significantly excited when spontaneous whisking occurred (green) and either significantly suppressed (ES, orange, left), not significantly modulated (En, orange, middle) or significantly excited (EE, orange, right) when sound-triggered whisking took place. Numbers in brackets indicate the number of neurons exhibiting a significant difference between the activity associated with spontaneous whisking and the activity associated with sound-triggered whisking according to receiver operating characteristic analysis (Fig. 5). ***B***, Mean fluorescence response for neurons that were significantly suppressed when spontaneous whisking occurred (green) and either significantly suppressed (SS, orange, left), not significantly modulated (Sn, orange, middle) or significantly excited (SE, orange, right) when sound-triggered whisking took place. The pie chart visualizes the proportions of neurons across the different categories. ***C***, Mean whisking event amplitude associated with spontaneous (green) and sound-triggered (orange) whisking. *N* = 2,209 spontaneous and 1,001 sound-triggered whisking events recorded in three mice. Shaded areas indicate 95% confidence intervals. ***D***, Neural response (mean of the *z*-scored fluorescence 0–1.5 s after whisking event) related to spontaneous whisking versus neural response to sound-triggered whisking. “R” indicates the Pearson’s correlation coefficient.

Of the 6,359 neurons recorded across 14 sessions in three mice 399, 6.3%, were significantly modulated (*p* < 0.001, Kruskal–Wallis test) by spontaneous whisking. The proportions were similar in A1 (6%, range across sessions: 0.5–13%) and AAF (9%, range: 1–15.5%). We then asked whether these neurons encoded sound-triggered whisking (Fig. 3*E*,*F*) in a similar way to spontaneous whisking. Surprisingly, many neurons responded very differently (Figs. 3*E*,*F*, 4*A*,*B*; Figs. S3, S1*D*), with 40% not significantly modulated by sound-triggered whisking despite being excited during spontaneous whisking (Fig. 4*A*, middle). This did not reflect a difference in the amplitude of sound-triggered whisking events, which were, in fact, slightly larger than the spontaneous whisker movements (Fig. 4*C*, *p* < 10^−24^, Wilcoxon rank sum test). It also did not reflect a decoupling between whisking and other externally observable indicators of arousal. Both whole-body twitching and pupil dilation also occurred with sound-triggered whisking (Fig. S4). Furthermore, there was a significant negative correlation between neural activity related to spontaneous whisking and neural activity related to sound-triggered whisking, suggesting that neurons which are strongly excited during spontaneous whisking tended not to be strongly activated by sound-triggered whisking (Fig. 4*D*).

The fact that pupil dilation (Fig. 1) and neuromodulatory signaling (Collins et al., 2023) co-occur with spontaneous whisking suggests an increase in arousal and the concomitant spike in neuromodulatory input to the cortex could be a potential driver of both spontaneous whisking and the change in neural activity associated with it. Because the relationship between whisking and neural activity is context dependent, often differing according to whether whisking occurred spontaneously or in response to sound, it is possible that this neuromodulatory signal is absent or different when whisking is sound triggered.

A potential driver of cortical modulation that occurs in relation to sound-triggered whisking (Fig. 4*A*,*B*, left and right) is the sounds themselves. However, only a small minority of neurons responded to tones of frequency-level combinations that did not cause whisking (54 of all 399 neurons and 20 of the 127 neurons that were significantly modulated during sound-triggered whisking). This shows that most neurons were not directly responsive to the tones used (although some may be tuned to frequency-level combinations that also elicit whisking). Furthermore, aligning the activity recorded after sound-triggered whisking to the sound onsets indicates that it occurs, on average, significantly later than sound-driven responses (Fig. S5) suggesting that the sounds are not generally the primary driver of that activity. Nevertheless, several factors suggest that what drives neural activity in relation to sound-driven whisking is different from what drives it when spontaneous whisking occurs, with direct acoustic stimulation being a likely candidate at least in some cases. First, large discrepancies between the whiskers' tuning to sound frequency and an individual neuron's tuning to sound frequency were sometimes observed (Fig. S6*A*). Second, timing and/or amplitude differences could be found between a neuron's response profile for spontaneous and sound-triggered whisking (Fig. S6*A,B*), including cases in which sound-triggered whisking and spontaneous whisking modulated neural activity in opposite directions (Fig. S6*C*; Fig. 4*A*, left, B, right). Ambiguity may remain in particular where a neuron exhibits no such differences and a close match exists between the neuron's tuning to sound frequency and the whiskers' tuning to sound frequency (Fig. S6*D,F*). Here, the most parsimonious explanation, other than the neuron exhibiting acoustic sensitivity that very closely matches its whisking sensitivity, is that the same driver is responsible for modulating its activity in relation to spontaneous and sound-triggered whisking.

**Figure 5.**
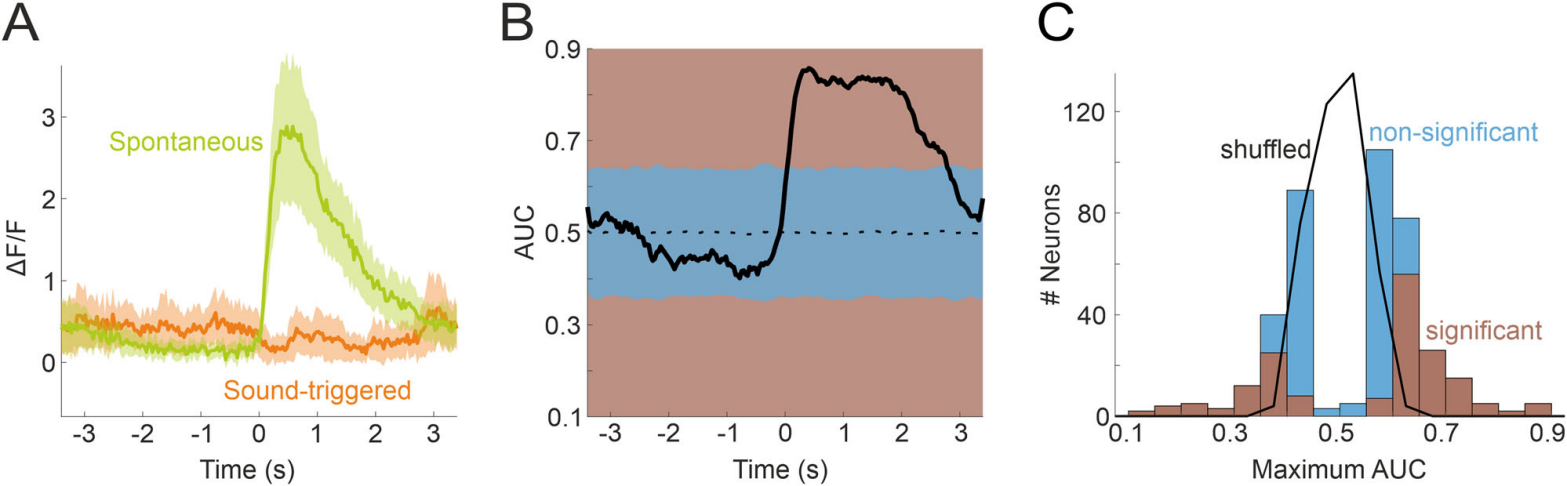
Receiver operating characteristic (ROC) analysis reveals neurons whose activity distinguishes between spontaneous and sound-triggered whisking. ***A***, Mean fluorescence response for one example neuron averaged across all spontaneous (green) and all sound-triggered (orange) whisking events. Time “0” indicates the timing of the whisking events. Shaded areas indicate 95% confidence intervals. ***B***, Area under the curve (AUC) as a function of time derived from ROC analysis applied to data shown in ***A***. Dotted line indicates mean AUC derived from ROC analysis applied to shuffled distributions. Blue area indicates 3 SD intervals around the mean AUC obtained with shuffled distributions. ***C***, Distribution of maximum AUCs (defined as maximum distance from 0.5) obtained for all neurons color coded according to whether the maximum AUC exceeded (brown, significant) the mean AUC obtained with shuffled distributions by 3 SDs or not (blue, nonsignificant). The black line indicates the distribution of maximum AUCs obtained with shuffled distributions.

To quantify differences between the neurons' response profiles for spontaneous and sound-triggered whisking, we applied a receiver operating characteristic (ROC) analysis to the distributions of neural activity measured around these events. An ROC curve was calculated separately for each time point (imaging frame), and the corresponding “area under the curve” (AUC; Fig. 5*A*,*B*) was considered significant if it exceeded the AUC of a control ROC curve obtained from shuffled distributions by 3 standard deviations (Gilad et al., 2020). Using this criterion, 175 of 399 (see numbers in brackets of Fig. 4*A*,*B* for proportions per category) neurons were deemed capable of distinguishing between spontaneous and sound-triggered whisking (Fig. 5*C*).

**Figure 6.**
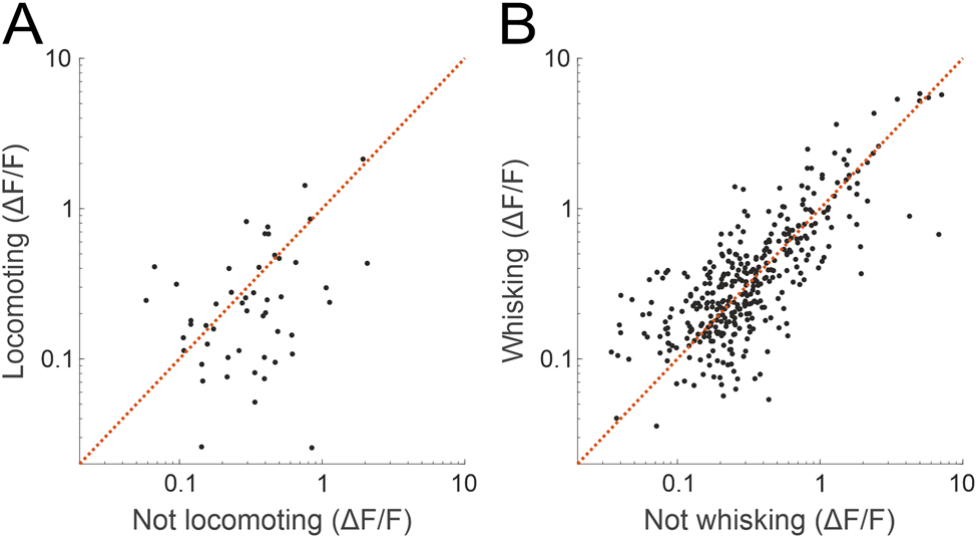
Locomotion suppresses sound-evoked activity in auditory cortex but whisking does not. ***A***, Mean sound-evoked activity to matching frequency-level combinations (see Materials and Methods) during locomotion versus stationary periods (median ± IQR = 0.17 ± 0.23 vs 0.34 ± 0.34 Δ*F*/*F*, *p* = 0.002, *n* = 59 neurons, Wilcoxon signed rank test). ***B***, Same as ***A*** for whisking versus not whisking (0.31 ± 0.37 vs 0.28 ± 0.37 Δ*F*/*F*, *p* = 0.14, *n* = 367 neurons, Wilcoxon signed rank test).

Movement, in particular locomotion, has been shown to affect sound-evoked activity in the auditory cortex. Therefore, we asked whether whisker movements also had an effect on sound-evoked neural activity. To this end, we compared tone responses that occurred when the mouse was whisking with responses to the same stimuli that occurred when the mouse was not whisking while disregarding any sounds that trigger whisking. In contrast to locomotion, which significantly suppressed sound-evoked neural responses (Fig. 6*A*), whisking had no effect (Fig. 6*B*).

## Discussion

Combining pupillometry with whisker and body movement tracking revealed that brief whisking bouts that happen spontaneously and independently of locomotion occur together with subtle, whole-body twitches and precede a dilation of the pupil that scales in size with the amplitude of the whisker movement. In addition, we observed that very similar whisking events can be triggered by presentation of sufficiently loud pure tones. In line with other reports linking orofacial movements with activity in sensory cortical areas (Musall et al., 2019; Stringer et al., 2019), including the auditory cortex (Clayton et al., 2024; Efron et al., 2025), we found that a subgroup of neurons in the auditory cortex was significantly modulated in relation to spontaneous whisking events. However, many of those neurons did not respond or responded differently to whisking events that were sound triggered. This indicates that we need to take into account the context (Brosch et al., 2005) and underlying drivers of a body movement in order to understand its relationship with auditory cortical activity.

### Arousal-related neuromodulation

The tight coupling between whisking and pupil diameter, combined with evidence that spontaneous whisker twitches signal a spiking in cholinergic and noradrenergic input to the cortex (Collins et al., 2023), suggests that the spontaneous whisking events that we recorded capture arousal-related shifts in neuromodulatory activity. Surges in neuromodulatory input to the cortex are, therefore, the most likely link between spontaneous whisking and the concomitant changes in auditory cortical activity. Auditory cortical neurons have been shown to carry strong signals related to pupil-indexed arousal (McGinley et al., 2015a,b; Schwartz et al., 2020; Saderi et al., 2021). This is evident not only from the large pupil dilations typically seen during states of hyperarousal and often occurring together with locomotion, but also with the small, brief dilations that we observed together with whisker twitching. These microdilations, signaling second-by-second fluctuations in arousal, have been shown to correlate closely with changes in the membrane potential of neurons in the auditory cortex (McGinley et al., 2015a).

Our finding that many auditory cortex neurons are modulated during spontaneous whisking but remain inactive or respond differently when whisking is triggered by sound suggests that the same arousal-related mechanisms are not responsible in each case. Sensory events, including sounds, can increase arousal, as evidenced by their ability to modulate pupil size (Bimbard et al., 2023; Turner et al., 2023; Clayton et al., 2024) and activate neuromodulatory centers like the basal forebrain, even if they have not been previously paired with reward or punishment (Guo et al., 2019; Robert et al., 2021; Zhu et al., 2023). However, the basal forebrain is not a uniform structure, with neural activity in the rostral basal forebrain correlating more closely with changes in pupil diameter than in the caudal part, whereas tone-evoked activity is stronger in the caudal basal forebrain (Robert et al., 2021). Additionally, the arousal-related neuromodulatory signal reaching the cortex is conveyed as a global, uniform signal, marked by widespread coordination that extends even across distant axons (Collins et al., 2023), whereas the sound-driven cholinergic input to the auditory cortex shows heterogeneous frequency tuning, which is dominated by a similar mid-to-low range that we found to be particularly effective at eliciting whisker movements and uncoupled from the tuning of nearby cortical neurons (Zhu et al., 2023). Stimulus-related activation of neuromodulatory structures would therefore not be expected to have the same downstream effects as activation during internally controlled brain state fluctuations. Indeed, the arousal increase prior to spontaneous locomotion and the arousal increase elicited by an air-puff have opposing effects on visual cortex activity (Vinck et al., 2015).

### Corollary discharge

Another potential driver of whisking-related activity in the auditory cortex is corollary discharge—copies of movement commands sent to sensory structures (Crapse and Sommer, 2008). In mice, the auditory cortex receives direct input from the secondary motor cortex (Nelson et al., 2013), though corollary discharge signals linked to whisking could also reach it indirectly, for example, via motor circuits projecting to the somatosensory cortex (Kleinfeld et al., 1999). Furthermore, stimulation of the primary somatosensory cortex can independently initiate whisking without involving the motor cortex (Matyas et al., 2010; Petersen, 2014), providing another potential source of corollary discharge signals. In addition, relevant motor centers may interface with the auditory pathway subcortically (Williamson et al., 2015; Yang et al., 2020).

Whisker movements are not only controlled by distinct neural circuits but also engage different populations of motor cortex neurons depending on what initiates the movement (Li et al., 2023). Consequently, movement signals received by auditory neurons during spontaneous whisking would likely differ from those related to sound-triggered whisking. Together with neuromodulatory signals linked to brain state, cortical and/or subcortical motor-auditory interactions (Lohse et al., 2022) are thus likely to shape how whisker movements are represented by the activity of auditory cortical neurons.

### Sound-triggered movements and neuroscience research

Consistent with other reports (Meyer et al., 2018; Bimbard et al., 2023; Clayton et al., 2024; Olsen and Hasenstaub, 2025), we found that sounds presented at moderate levels reliably elicit movements in mice, presumably by engaging motor and somatosensory circuits and altering the animal's brain state. This has important practical implications not only for auditory, somatosensory and multisensory research, but also in the many behavioral studies where acoustic stimuli are used as cues. While the recent detailed characterization of sound-triggered facial movements by Clayton et al. (2024) offered the reassuring conclusion that narrowband sounds like pure tones pose no issues as long as they are presented below 90 dB SPL, our data suggest that this recommendation may require some refinement. We found that pure tones at frequencies within the most sensitive region of the mouse's hearing range elicited whisking in almost every trial when presented at 80 dB SPL and triggered measurable and more narrowly tuned whisker movements at levels as low as 50 dB SPL. These level-dependent changes in the frequency tuning of whisker movements resemble the broadening in frequency selectivity observed in the mouse auditory periphery (Song et al., 2008; Dewey and Shera, 2023).

The mice employed in our study had a regular C57BL/6 background, a strain that is prone to age-related hearing loss, and would have been expected to show early indications of high-frequency loss at the time of data collection (Ison et al., 2007; Zhang et al., 2013; Johnson et al., 2017). In other mouse strains, or younger animals, pure tone stimulation might be even more effective at triggering movement than reported here, especially at higher frequencies. The most likely reason for why we observed sound-triggered movement at lower tone intensities than Clayton et al. (2024) is that we used longer sound duration tones (200 ms vs 50 ms). In order to avoid potential confounds arising from sound-triggered body movements, we would recommend presenting low intensity, short duration sounds and/or avoid frequencies to which mice are particularly sensitive. Where the scientific question requires the stimulus space to be sampled more comprehensively, it can be beneficial to carry out control experiments in anesthetized animals in order to disentangle acoustic and nonacoustic drivers of activity.

It will be important in future studies to establish whether the decoupling of neural representations for spontaneous and sound-triggered movements originates in the cortex or is already evident subcortically, whether it extends across different types of sounds, and to what extent it can be accounted for by neuromodulatory activity.
